# Severe bronchial reaction to provocation with fish and crustaceans 

**DOI:** 10.5414/ALX02129E

**Published:** 2020-12-02

**Authors:** Alexandra M. Preisser, Alexander M. Kraft, Volker Harth

**Affiliations:** Institute for Occupational and Maritime Medicine (ZfAM), University Medical Center Hamburg-Eppendorf, Hamburg, Germany

**Keywords:** occupational asthma, type I sensitization, fish, crustacean, specific inhalation challenge (SIC)

## Abstract

The specific inhalation challenge (SIC), a workplace-related inhalation exposure test, is used to identify allergic asthma when symptoms such as coughing, wheezing, or dyspnea occur at the workplace. Its use is risky. A cook (28 years old) has been complaining of rhinoconjunctivitis and contact urticaria while preparing seafood for 3 years. He continues to work, now wears gloves, no longer tastes fish dishes, and receives anti-obstructive therapy (ICS, LABA). Methacholine (MCH) testing for bronchial hyperreactivity (BHR) shows mild BHR (PD_100;sRaw_: 0.28 mg MCH), skin and blood tests show type I sensitization to fish and crustacean proteins. In SIC with fried shrimps, rhinoconjunctivitis, coughing and distance wheezing, FEV_1_ drop > 20%, sRaw increase to 9.6 kPa*s and angioedema occur. Since routine tests showed only a moderate BHR, the suspicion of an occupational disease was formulated very late in the medical examination process. Only the SIC showed the severity of the cook’s bronchial asthma.

[Table Abbreviation]

**German version published in Allergologie, Vol. 43, No. 1/2020, pp. 20-25**

## Introduction 

The “specific inhalation challenge” (SIC) is a valuable diagnostic tool for identifying allergic and immunological asthma in the assessment of work-related obstructive airway symptoms when the results of the occupational history, allergological examinations, and pulmonary function test are ambiguous [1]. The SIC aims to reproduce exposures under laboratory conditions comparable to the patient’s work [2]. SIC testing is also generally considered to be the reference standard to identify and document the clinical relevance of new agents for the upper and lower respiratory tract. It makes it possible to determine the probable causal relationship between workplace-related inhalation exposure and respiratory tract or lung disease and is thus an important element in answering the question of whether the patient should continue his or her professional activity [3]. Despite its great benefits, the SIC also involves risks. 

We report about a cook in whom the severity of bronchial asthma in the sense of an occupational disease only became apparent in the SIC. 

## Case history 

A highly qualified chef (28 years old) of an à-la-carte restaurant presented to our occupational and environmental medicine outpatient clinic of the Institute for Occupational Medicine and Maritime Medicine, a department of the University Medical Center Hamburg-Eppendorf with the question whether an occupational disease should be recognized by the German Social Accident Insurance (DGUV). Three years earlier he had already noticed skin redness and swelling when preparing fish and crustaceans. Over the years, the symptoms progressed to include conjunctival irritation, cough, and sometimes shortness of breath. The skin reactions were, as he reports, particularly severe when filleting fish, e.g., cod but also other species. Protective gloves would only alleviate the skin symptoms slightly. Shortness of breath and cough, combined with conjunctivitis, would occur especially if crustaceans such as scampi or shrimps were fried in the kitchen. He then used salbutamol as a short-acting inhaled β-2 sympathomimetic (SABA) in addition to the long-term inhaled therapy stage III (inhaled corticosteroid (ICS) medium dose, long-acting inhaled β-2 sympathomimetic (LABA)). Because of the respiratory distress he had repeatedly been unable to work. He tries to delegate the work with the food causing the symptoms, but this is often not practicable. Due to these restrictions, he has no career chances, because as a chef in a higher and leading position he has to at least taste the fish and crustacean dishes. 

He started his apprenticeship 10 years ago in a fish restaurant. At that time, he had already noticed respiratory problems, which is why he had consulted his GP. The physician had attributed the complaints to a grass and pollen allergy. He had to interrupt his training for personal reasons and initially joined the military for 23 months. Afterwards, he continued his training as a cook and successfully completed it. He worked in high-class hotels for 7 years. Four years ago, shortly before the end of the training, he once suffered an anaphylactic reaction with shortness of breath after tasting smoked fish but was eager to complete his training. Specific IgE antibodies to shrimp, fish, and seafood mixes were detected and he has since owned an allergy passport; however, an occupational disease report was not submitted to the Employer’s Liability Insurance Association. He then continued his career in Austria and Germany working as a “Commis de cuisine” and then as a “Demi Chef de Partie” in high-quality restaurants or as a self-employed chef. 

In childhood, “constricted bronchial tubes” had been diagnosed, but asthma had never been confirmed. In spring and early summer, seasonal runny nose and sneezing occur, but there is no shortness of breath. 

### Findings

The 28-year-old man (non-smoker for 3 years, previously 7 pack years) was in good general condition and had normal weight (89 kg, 184 cm). Clinically, the heart, lungs, and upper respiratory tract were inconspicuous. Long-term medication with ICS/LABA combination p.i. 2 × daily and SABA as needed was paused 2 weeks before the examinations. The airway resistance was slightly increased (sRaw 1.23 kPa*s) with normal FEV_1_ (95% of pred. value) when therapy was suspended. The methacholine (MCH) test showed a mild bronchial hyperreactivity (PD_100;sRaw_: 0.28 mg MCH). In the prick test, histamine subthreshold reactions to house dust mites, storage mites, and grass mix were found. Blood tests showed type I sensitization to various fish and crustacean proteins (cod, trout, salmon, sole, shrimp, lobster, and crawfish) at a total IgE of 111 kU/l with IgE CAP classes of 3 – 4; specific IgE antibodies to the recombinant allergen of shrimp rPen a 1 (tropomyosin) were determined at 12.0 kU/L corresponding to CAP class 3. All other laboratory findings were normal except for neutrophilic leukocytosis of 11.5 c/nL. 

## Specific inhalation challenge 

**Figure 1:**
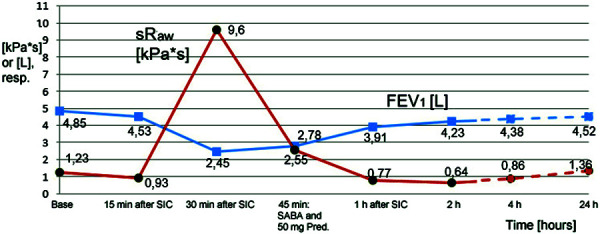
Lung function measured values of FEV1 and specific airway resistance (sRaw) within the scope of the SIC (FEV_1_ predicted value according to GLI: 4.9 L).

**Figure 2:**
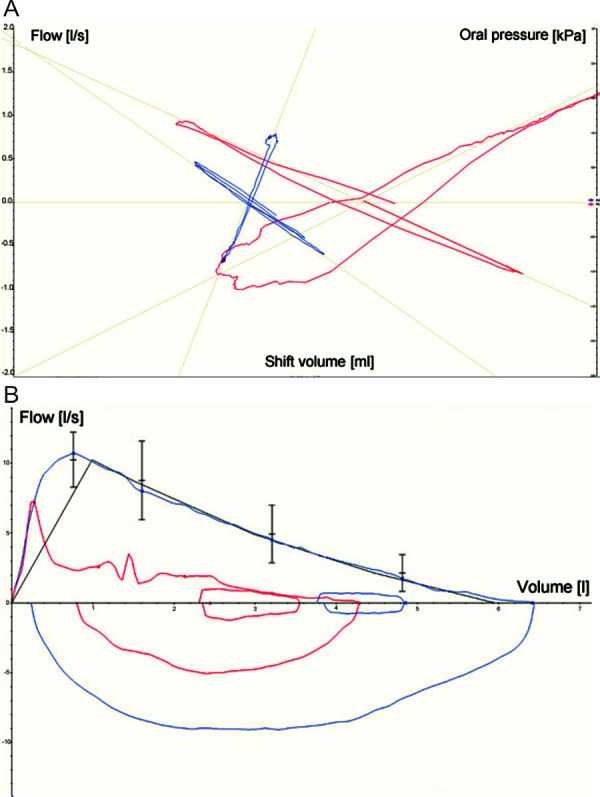
A: Body plethysmography and B: Spirometry: baseline measurement (blue) and 15 minutes after the start of specific inhalation exposure to shrimp (red).

The situation at the workplace was simulated as closely as possible in the test. For this purpose, the cook brought the necessary materials, freshly caught cod and fresh prawns. The fish and crustaceans were processed in a kitchen with a cutting board and hot plate, which was simulated at our institute. Filleting of the cod and peeling of the shrimps already led to contact urticaria, initially, however, without dyspnea and without altering the lung function values. During frying of the shrimps, rhinoconjunctivitis, coughing, and distance wheezing, angiedema of the face, and ubiquitous pruritus occurred. FEV_1_ decreased by more than 20%, sRaw increased 8-fold to 9.6 kPa*s; thus, the positive criteria of the SIC (FEV_1_ decrease of at least 20% from baseline, doubling of sRaw and increase > 2 kPa*s) were met. The inhalation of 3 salbutamol doses of 100 µg each caused a rapid improvement of the obstruction (Figure 1 and Figure 2); for the persistent general symptoms with pruritus, 50 mg prednisolone was additionally administered orally once. 

## Discussion 

Fish and crustacean allergies are not uncommon in cooks, and the development of severe allergic symptoms is possible. In the reported case of a highly qualified chef, only mild bronchial hyperreactivity was initially observed; the severity of bronchial asthma with angioedema in the sense of an anaphylactic reaction only became evident in the later SIC [4]. The main allergen in cod, parvalbumin (Gad c 1), is known to be very heat-stable and not only remains stable during the cooking process, but can also pass into the air (e.g., into the cooking steam). It cannot be destroyed by chemical cleaning agents either. 

Although the SIC represents a complex diagnostic procedure, it can – as in the case described – make a crucial contribution to the diagnosis, which could not be confirmed by patient history and clinical findings alone. However, due to the possibility of a severe bronchial or anaphylactic reaction and the related risk for the patient, a strict indication for the use of the SIC must be applied [1]. 

The SIC is indicated when there is clinical evidence of work-related asthma and/or an occupational disease (in Germany BK 4301, 4302, 1315, or 4201), but available information is not sufficient to confirm the diagnosis. The SIC can also be used to derive important therapeutic or preventive measures at the workplace or in individual prevention (e.g., change of profession). It is rarely indicated in the diagnosis of unclear workplace-related respiratory problems that cannot be narrowed down further by patient history and allergy diagnostics [3]. 

SIC is contraindicated if the diagnosis can be made with an adequate accuracy for the specific problem using simpler diagnostic measures [3]. In the reported case this was not clear, and the association between exposure and symptoms did not lead the patient to change his profession. The SIC led to the recognition of the occupational disease and he began retraining for a commercial profession. Individual factors must also be considered as contraindications, such as a pre-existing higher-grade airway obstruction. The obstruction often only becomes apparent when the anti-obstructive and anti-inflammatory medication is discontinued, which is necessary for the proper performance of the SIC. The SIC should be performed without anti-obstructive therapy to avoid false negative test results. 

To minimize the time without medication, in the present case the dermal exposure with fish and the inhalation provocation were performed directly one after the other. This can intensify the symptoms, e.g., by delayed reactions. The international guidelines recommend that the SIC be conducted with the various agents over several days [5]. Running the test on different days could increase the information about the triggering allergen. However, this is not mandatory for the confirmation of an occupational disease. However, it is usually not practical or reasonable to carry out the test over several days. In addition, discontinuation of the medication could increase the obstruction, thwart the test, and endanger those affected. 

SIC involves the risk of an anaphylactic reaction that may manifest not only in the respiratory tract but can also affect the skin, abdomen, or cardiovascular system. These organ systems must therefore be monitored after the SIC is performed, or at least symptoms must be controlled. According to the classification of the severity of anaphylaxis by Ring and Messmer, the patient in the case study had a moderately severe grade (grade II of a maximum of IV) [6]. In addition to anti-obstructive therapy, the patient was treated with systemically administered cortisone. It should be critically noted that the primary therapy of an anaphylactic reaction would have been the administration of adrenaline [4], e.g., as an intramuscular administration with an emergency pen. The administration of adrenaline can reduce the risk of a biphasic reaction [7]; there is no evidence for the administration of cortisone. Delayed reactions, however, are usually of only mild to moderate symptom severity and occur mostly within 8 hours [7]. 

The notification as an occupational disease was delayed in the described case. Already 3.5 years earlier, an allergic general and respiratory reaction after tasting fish could have been the occasion to report the substantiated suspicion of an occupational disease. The development of bronchial asthma requiring therapy could possibly have been reduced; at least the imminent change of profession would have taken place earlier. To make it easier to check whether a report on a suspected allergic occupational disease (in Germany BK 4301) should be submitted, the German Social Accident Insurance (DGUV) has created an online help in which the most frequent occupational allergens are listed that can trigger an allergic rhinopathy or allergic bronchial asthma, see box [Table Box][8]. 

## Essential sentence 

The notification of workplace-related asthma must be made at an early stage if an occupational disease is suspected. The specific inhalation challenge (SIC) is well suited to detect the consequences of occupational exposure but carries the risk of a severe bronchial and systemic allergic reaction. 

## Funding 

None. 

## Conflict of Interest 

Alexandra M. Preisser, Alexander M. Kraft and Volker Harth declare that they have no conflict of interest. 

**List of abbreviations Abbreviation:** List of abbreviations.

BHR	Bronchial hyperreactivity
BK	Occupational disease
CAP	Carrier-Polymer-System (test system for the determination of IgE)
DGUV	German Social Accident Insurance
FEV_1_	Forced expiratory volume in 1 second
GLI	Global Lung Initiative
GP	General Practitioner
ICS	Inhaled corticosteroid
IgE	Immunoglobulin E
kPa*s	Kilopascal x second
LABA	Long-acting inhaled β-2 sympathomimetic
MCH	Methacholine
PD100	Provocation dose that causes a 100% increase
SABA	Short-acting inhaled β-2 sympathomimetic
SIC	Specific inhalation challenge
sRaw	Specific airway resistance
TRBA	Technical rules for biological agents
TRGS	Technical rules for hazardous substances

**Box Box:** Causes of occupational allergic rhinopathy or bronchial asthma that warrant a suspected occupational disease report (examples, [8]):

–**Herbal allergens:** dust of flour and bran from cereals, dust of various types of timber, castor oil bean dust, green coffee bean dust, cocoa bean dust, lycopodium dust, aerosols containing mold, bell pepper pollen, strawberry pollen, tomato pollen, feed dust, tea dust.
–**Livestock allergens:** insect dust, feather dust, hair dust, raw silk dust, mother-of-pearl dust, mites, urine from rodents, animal epithelia.
–**Miscellaneous allergens:** Substances classified as respiratory tract-sensitizing in the safety data sheet or in the commercial register of hazardous substances (see also TRGS 406 [9]), e.g., antibiotics, sulfonamides, proteases, and enzyme-containing dusts (e.g., α-amylase, phytase), platinum, persulfates.
